# Supplementary material for: Getting More Out of Biomedical Documents with GATE's Full Lifecycle Open Source Text Analytics
Source: PLoS Comput Biol. 2013 Feb 7;9(2):e1002854. doi: 10.1371/journal.pcbi.1002854 (PMC3567135; doi:10.1371/journal.pcbi.1002854)
Supplement: Dataset S1 — GATE software. Dataset S1 bundled with this paper contains a distribution of GATE (or see http://gate.ac.uk/download/). (TGZ) [file pcbi.1002854.s001.tgz › plos-gate/doc/announce-4-0.html]

GATE Version 4.0 release (July 2007)


# GATE Version 4.0 release (July 2007)

# 1. Major new features

## 1.1. ANNIC

ANNotations In Context: a full-featured annotation indexing and retrieval
system designed to support corpus querying and JAPE rule authoring. It is
provided as part of an extention of the Serial Datastores, called Searchable
Serial Datastore (SSD)
(details).

## 1.2. New machine learning API

A brand new machine learning layer specifically targetted at NLP tasks
including text classification, chunk learning (e.g. for named entity
recognition) and relation learning
(details).

## 1.3. Ontology API

A new ontology API, based on OWL In Memory
(OWLIM), which offers a better API, revised ontology event model and
an improved ontology editor to name but few
(details).

## 1.4. OCAT

Ontology-based Corpus Annotation Tool to help annotators to manually annotate
documents using ontologies
(details).

## 1.5. Alignment Tools

A new set of components (e.g. CompoundDocument, AlignmentEditor etc.) that help
in building alignment tools and in carrying out cross-document processing
(details).

## 1.6. New HTML Parser

A new HTML document format parser, based on Andy Clark's
NekoHTML. This parser
is much better than the old one at handling modern HTML and XHTML constructs,
JavaScript blocks, etc., though the old parser is still available for existing
applications that depend on its behaviour.

## 1.7. Java 5.0 support

GATE now requires Java 5.0 or later to compile and run. This brings a number
of benefits:

- Java 5.0 syntax is now available on the right hand side of JAPE rules
  with the default Eclipse compiler
  (details).
- enum types are now supported for resource parameters. see
  here for details on
  defining the parameters of a resource.
- AnnotationSet and the CreoleRegister take advantage of
  generic types. The AnnotationSet interface is now an extension of
  Set<Annotation> rather than just Set, which should make for
  cleaner and more type-safe code when programming to the API, and the
  CreoleRegister now uses parameterized types, which are
  backwards-compatible but provide better type-safety for new code.

# 2. Other new features and improvements

- Hiding the view for a particular resource (by right clicking on its tab
  and selecting "Hide this view") will now completely close the associated
  viewers and dispose them. Re-selecting the same resource at a later time will
  lead to re-creating the necessary viewers and displaying them. This has two
  advantages: firstly it offers a mechanism for disposing views that are not
  needed any more without actually closing the resource and secondly it
  provides a way to refresh the view of a resource in the situations where it
  becomes corrupted.

- The DataStore viewer now allows multiple selections. This lets users load
  or delete an arbitrarily large number of resources in one operation.

- The Corpus editor has been completely overhauled. It now allows
  re-ordering of documents as well as sorting the document list by either index
  or document name.

- Support has been added for resource parameters of type
  gate.FeatureMap, and it is also possible to specify a default value
  for parameters whose type is Collection, List or
  Set (details).

- (Feature Request
  1446642)
  After several requests, a mechanism has been added to allow overriding of
  GATE's document format detection routine. A new creation-time parameter
  mimeType has been added to the standard document implementation, which
  forces a document to be interpreted as a specific MIME type and prevents the
  usual detection based on file name extension and other information
  (details).

- A capability has been added to specify arbitrary sets of additional
  features on individual gazetteer entries. These features are passed forward
  into the Lookup annotations generated by the gazetteer
  (details).

- As an alternative to the Google plugin, a new plugin called *yahoo* has been
  added to GATE to allow users to submit their query to the Yahoo search engine
  and to load the found pages as GATE documents
  (details).

- It is now easier to run a corpus pipeline over a single document in the
  GATE GUI -- documents now provide a right-click menu item to create a
  singleton corpus containing just this document
  (details).

- A new interface has been added that lets PRs receive notification at the
  start and end of execution of their containing controller. This is useful
  for PRs that need to do cleanup or other processing after a whole corpus has
  been processed
  (details).

- The GATE GUI does not call System.exit() any more when it is closed.
  Instead an effort is made to stop all active GATE threads and to release all
  GUI resources, which leads to the JVM exiting gracefully. This is
  particularly useful when GATE is embedded in other systems as closing the
  main GATE window will not kill the JVM process any more.

- The set of AnnotationSchemas that used to be included in the core
  gate.jar and laoded as builtins have now been moved to the ANNIE plugin.
  When the plugin is loaded, the default annotation schemas are instantiated
  automatically and are available when doing manual annotation.

- There is now support in creole.xml files for automatically creating
  instances of a resource that are hidden (i.e. do not show in the GUI). One
  example of this can be seen in the creole.xml file of the ANNIE plugin where
  the default annotation schemas are defined.

- A couple of helper classes have been added to assist in using GATE within
  a Spring application
  (details).

- Improvements have been made to the thread-safety of some internal GATE
  components, which mean that it is now safe to create resources in
  multiple threads (though it is not safe to use the same resource instance in
  more than one thread). This is a big advantage when using GATE in a
  multithreaded environment, such as a web application
  (details).

- Plugins can now provide custom icons for their PRs and LRs in the plugin
  JAR file (details).

- It is now possible to override the default location for the saved session
  file using a system property
  (details).

- The TreeTagger plugin supports a system property to specify the location
  of the shell interpreter used for the tagger shell script. In combination
  with Cygwin this makes it much easier to use the tagger on Windows
  (details).

- The Buchart plugin has been removed; it is superseded by SUPPLE. The
  probability finder plugin has also been removed, as it is no longer
  maintained.

- The bootstrap wizard now creates a basic plugin that builds with Ant.
  Since a Unix-style make command is no longer required this means that the
  generated plugin will build on Windows without needing Cygwin or MinGW.

- The GATE source code has moved from CVS into Subversion. See
  here for details of how to check
  out the code from the new repository.

- An optional parameter, keepOriginalMarkupsAS, has been added to the
  DocumentReset PR which allows users to decide whether to keep the Original
  Markups AS or not while reseting the document
  (details).

# 3. Bug fixes and optimizations

- The Morphological Analyser has been optimized. A new FSM based, although
  with minor alteration to the basic FSM algorithm, has been implemented to
  optimize the GATE Morphological Analyser. The previous profiling figures show
  that the morpher when integrated with ANNIE application used to take upto
  60% of the overall processing time. The optimized version only takes 7.6%
  of the total processing time.
  (details).

- The ANNIE Sentence Splitter was optimised. The new version is about twice
  as fast as the previous one. The actual speed increase varies widely
  depending on the nature of the document.

- The implementation of the *OrthoMatcher* component has been
  improved. This resources takes significantly less time on large documents.

- The implementation of AnnotationSets has been improved. GATE now
  requires up to 40% less memory to run and is also 20% faster on average.
  The get methods of AnnotationSet return instances of
  ImmutableAnnotationSet. Any attempt at modifying the content of
  these objects will trigger an Exception. An empty
  ImmutableAnnotationSet is returned instead of null.

- The Chemistry tagger has been updated with a number of bugfixes and
  improvements
  (details).

- The Document user interface has been optimised to deal better with large
  bursts of events which tend to occur when the document that is currently
  displayed gets modified. The main advantages brought by this new
  implementation are:
  - The document UI refreshes faster than before.
  - The presence of the GUI for a document induces a smaller performance
    penalty than it used to. Due to a better threading implementation, machines
    benefiting from multiple CPUs (e.g. dual CPU, dual core or hyperthreading
    machines) should only see a negligible increase in processing time when a
    document is displayed compared to the situations where the document view is
    not shown. In the previous version, displaying a document while it was
    processed used to increase execution time by an order of magnitude.
  - The GUI is more responsive now when a large number of annotations are
    displayed, hidden or deleted.
  - The strange exceptions that used to occur occasionally while working
    with the document GUI should not happen any more.

And as always there are many smaller bugfixes too numerous to list here...
